# The novel anti-cancer feature of Brazzein through activating of hTLR5 by integration of biological evaluation: molecular docking and molecular dynamics simulation

**DOI:** 10.1038/s41598-022-26487-2

**Published:** 2022-12-20

**Authors:** Maede Poursalim, Marzieh Dehghan Shasaltaneh, Vahab Jafarian, Hafezeh Salehabadi

**Affiliations:** 1 Department of Biology, Faculty of Sciences, University of Zanjan, Zanjan, Iran; 2 Department of Medicinal Chemistry, School of Pharmacy, Zanjan University of Medical Sciences, Zanjan, Iran

**Keywords:** Cancer, Computational science

## Abstract

Many of plant proteins exhibit the properties similar to the antitumor proteins although the anticancer activity of Brazzein on modulating the autophagy signaling pathway has not been determined so far. The present study aimed to develop a simplified system to enable the rational design of the activating extracellular domain of human Toll-like receptor 5 (hTLR5). To identify the anticancer effect of Brazzein, HADDOCK program and molecular dynamics (MD) simulation were applied to examine the binding of the wild type (WT) and p.A19K mutant of Brazzein to the TLR5. The expression of *MAP1S* and *TNF-α* genes was estimated based on real-time PCR. The results clearly confirmed that the WT of Brazzein activated hTLR5 in the MCF-7 cell line since the genes were more and significantly less expressed in the cells treated with the WT and p.A19K mutant than the control, respectively. The snapshots of MD simulation exhibit the consistent close interactions of hTLR5 with the two helices of Brazzein on its lateral side. The results of per residue-free energy decomposition analysis substantiate those of intermolecular contact analysis perfectly one. We propose that the WT of Brazzein can act as an antitumor drug candidate.

## Introduction

Breast cancer is considered as a non-communicable disease among women, which is the leading cause of their cancer death^1^. Common treatments for cancer such as chemotherapy, and radiology consistently exert different effects on patients. The appropriateness of food-derived bioactive peptides like anticancer (ACPs) and antimicrobial peptides (AMPs) as cancer treatment options has been proven scientifically. Based on the results of the recent studies, some characteristics of bioactive peptides such as small size, easy manufacture, naturalness, effectiveness, and minimum side effects, as well as an improved effect of cell membranes make them better options in cancer therapy^2^. In addition, the majority of ACPs have similar properties to AMPs^3^. AMPs are usually short peptides with 12–100 residues, which are mainly positively charged among which some are negatively- and neutrally-charged molecules. Most of the plant AMPs having anticancer activity possess a molecular weight of 2–10 kg/mol (kDa), and contain 4, 6, 8, or 12 cysteine (Cy) residues which form disulfide bonds^4^. The features of many of the proteins extracted from plants are similar to those of anticancer proteins.

Further, Brazzein protein is isolated from the fruit of *Pentadiplandra brazzeana Baillon*, composing of the three strands of antiparallel β-sheet, as well as a short α-helix connected by loops. It consists of 54 residues and has a molecular weight of about 6.5 kDa, in the structure of which eight Cys residues generate four disulfide bonds between the beta-sheets^5,6^. The protein is predicted to exhibit antibacterial and antifungal properties due to specific structural characteristics. Furthermore, some researchers have reported the antioxidant, anti-allergic, and anti-inflammatory effects of Brazzein. However, the biological activity of the protein has not been understood fully^6^. Regarding the Brazzein, the loops are mostly on the surface, at which the p.A19K mutation (point mutation) is located. The mutant is a positive charge which can more interact with its environment^5,6^.

The innate immune system is considered as important in treatment with exogenous proteins, which identifies ligands using several classes of receptors, commonly known as pattern recognition receptors (PRRs). The Toll-like receptor (TLR) family was the first detected PRRs^7^. TLR ligands are categorized into exogenous and endogenous, the first of which contains the pathogen-associated molecular patterns (PAMPs) such as the proteins, lipoproteins, lipids, and nucleic acids obtained from bacteria, viruses, parasites, and fungi. However, the endogenous ligands include the damage-associated molecular patterns (DAMPs) like high-mobility group box 1 (HMGB1), DNA, and heat shock proteins 60, 70, and 90^8,9^. TLRs can signal via one or both of Myeloid differentiation factor 88 (MyD88)-dependent pathway and -independent (TRIF-dependent) one^10,11^.

Additionally, the expression of TLRs has been reported in the types of immune cells such as monocytes, T and B lymphocytes, mast, myeloid dendritic (DCs), and fibroblast-like cells, and epithelial cells in humans^12^. The results of the previous studies confirmed the expression of TLR2, TLR5, and TLR6 in MCF-7 cell line^13^. TLR5 is highly expressed in breast cancer and its signaling plays a crucial role in the cancer. It is among TLRs that pathogen-associated molecular pattern is primarily a protein. The TLR5 ligand flagellin can induce signaling through MyD88 adapter protein, leading to NF-κB activation and more inflammatory cytokine transcription^7,14^. TLR signaling in cancer promotes cancer cells or tumor cell apoptosis, or even inhibits their growth. TLR5 activation enhances antitumor properties and represses an increase in breast cancer cells^15^. In the breast cancer, TLR5 activation leads to autophagy via the *MAP1S* autophagy protein which induces IL-8 and TNF-α in the MCF-7 cell line. The induction of TLR5 signaling by autophagy is a delayed response, which takes approximately 16 h^16^. *MAP1S* is implicated in the dynamics of microtubules, mitotic abnormalities, and cell death^17^. There is little information in functionality of TLR5 in cancer. Despite the similarity between the characteristics of many plant proteins and anticancer ones, the anticancer effect of Brazzein on modulating the autophagy signaling pathway remains unclear. In the present study, a simplified system was developed to allow the design of the activating extracellular domain of Toll-like receptor 5 (hTLR) rationally. Leucine-rich repeat (LRR) proteins 7 and 9 can be considered among large surface areas, which only serve at a single site covering a small surface area through interacting with Brazzein with high affinity. The results of the study suggested the ability of Brazzein to act as an anticancer peptide due to its structure, sequence, and special properties, and subsequently probable interaction of the protein with TLR5. In silico studies are able to produce various datatypes, and offer a complementary approach to in vivo observations. They are being used to evaluate the mechanism of the interaction between the ligands and macromolecules. The results can be used to designing the experimental procedures, explaining the experimental findings as well as treatment purposes^18–21^.

This study for the first time assessed the antitumor activity of Brazzein on the MCF-7 cell line in vitro and in silico to the best of our knowledge.

## Results

### Quality of constructed model

The homology modeling results of the study, were reviewed to select the best developed model for other processes. In addition, the overall quality of the model was further examined by using the parameters of Verify 3D, Z-DOPE, and RMSD (Table 1). The 3D model was accepted according to the standard values for the parameters (RMSD < 1 Å, Z-DOPE $\le$ − 1, and Verify 3D = 80–100%).

**Table 1:** Results of p.A19K Brazzein model.

Parameters	RMSD	Z-DOPE	Verify 3D (%)
p.A19K	0.32	− 1.015	96.23

### Protein–protein interaction (PPI)

The interactions between either the WT or mutant with hTLR5 were assessed through applying the molecular docking using a HADDOCK algorithm^22^. Table 2 lists the free energy of docking values for the WT- and mutant-hTLR5 complexes. Based on the results, the minimum Z-score was observed in the WT, reflecting that the p.A19K substitution can destabilize the brazzein-hTLR5 complex (Table 2). Thus, MD simulation was employed to confirm this hypothesis.

**Table 2:** Cluster statistics of the HADDOCK docking run calculated on the top 4 members of each cluster.

Parameters	Wild-type	p.A19K
HADDOCK score (a.u)^a^	− 141.2 ± 1.5	− 130.5 ± 12
Cluster size	23	8
RMSD from overall lowest energy structure (Å)	0.9 ± 0.5	0.7 ± 0.4
Intermolecular van der Waals energy (Evdw)^b^ (Kcal mol^−1^)	− 60.3 ± 5.5	− 69.3 ± 0.9
Intermolecular electrostatic energy (Eelec)^b^ (Kcal mol^−1^)	− 434.3 ± 24.7	− 294.1 ± 44.7
Desolvation energy (Edesol)^c^ (Kcal mol^−1^)	− 33.8 ± 8.9	− 37.0 ± 10.1
Restraints violation energy (EAIR) (Kcal mol^−1^)	398.2 ± 73.65	345.3 ± 57.11
Buried surface area (BSA)	2093.3 ± 69.1	1902.6 ± 31.6
Z-score	− 2.2	− 2.5

*RMSD* root mean squared deviation, *A19K* Alanine substitution conserved Lysine at residue 19.

^a^The HADDOCK score is defined as: 1.0 E_vdw_ + 0.2 E_elec_ + 1.0 E_desol_ + 0.1 E_AIR_.

^b^Non-bonded interactions were calculated with the OPLS force field using a 8.5 Å cut-off.

^c^Calculated using the empirical desolvation energy parameters.

**Figure 1:**
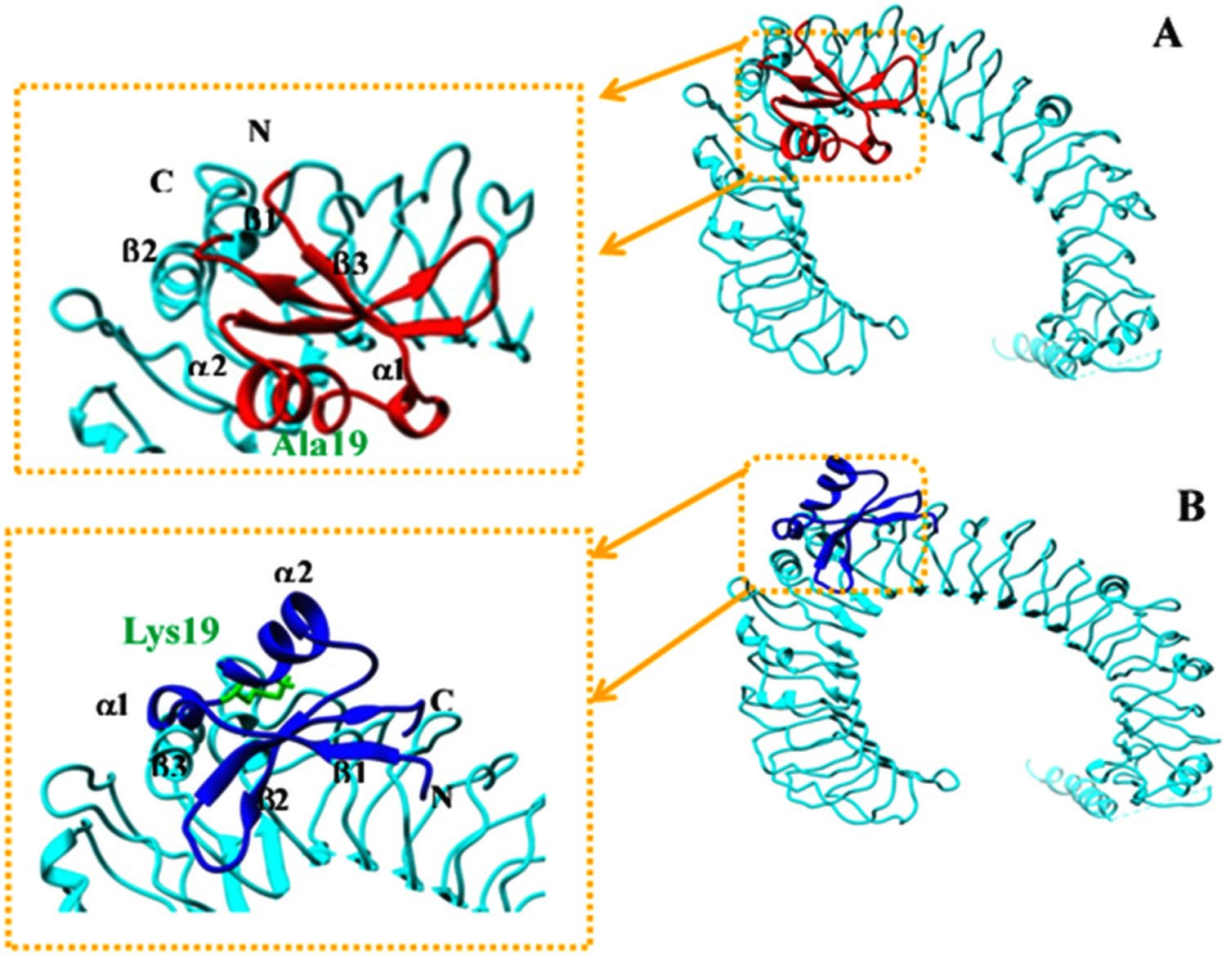
Solution NMR structures of the Brazzein with hTLR5. (**A**) wild-type (WT) of Brazzein interacts with near concave β-sheet domain of hTLR5. (**B**) The substitution Ala 19 with Lys near the α2-helixes sites of Brazzein bond to hTLR5. The N- and C-terminals are denoted by N- and C-, respectively.

The 3D structure of the favorable complex is displayed in Fig. 1. As shown, the concave β-sheet domains of TLR5 and conserved α2-helixe sites of Brazzein form the binding interface, leading to higher interface distortion and more buried residues, which strengthens the binding of the two proteins. Further, Ala substitution to Lys reverses the structure of Brazzein in the binding pocket of hTLR5 (Fig. 1A,B), which is likely to unstabilize the interaction between p.A19K mutant and hTLR5. The subsequent analyses could confirm this hypothesis.

### Effects of mutation on the binding pocket size

CASTp server measures all surface and internal pockets, and their exact volume and area, as well as the size of openings^23^. Before the mutation, the area of the binding pocket 1 was equal to 178.725, which elevated to 2351.578 following the mutation and covered almost the entire molecule (Table 3).

**Table 3:** Predict the effect of mutation on the binding pocket area “before and after mutation” by the CASTp server.

Parameters	Wild-type	p.A19K	Difference amount
Pocket ID	1	1	–
Area (SA)	178.725	2351.578	+ 2172.853
Volume (SA)	57.251	30,511.658	+ 30,454.407

*SA* shaded area.

### MD simulation analysis

**Figure 2:**
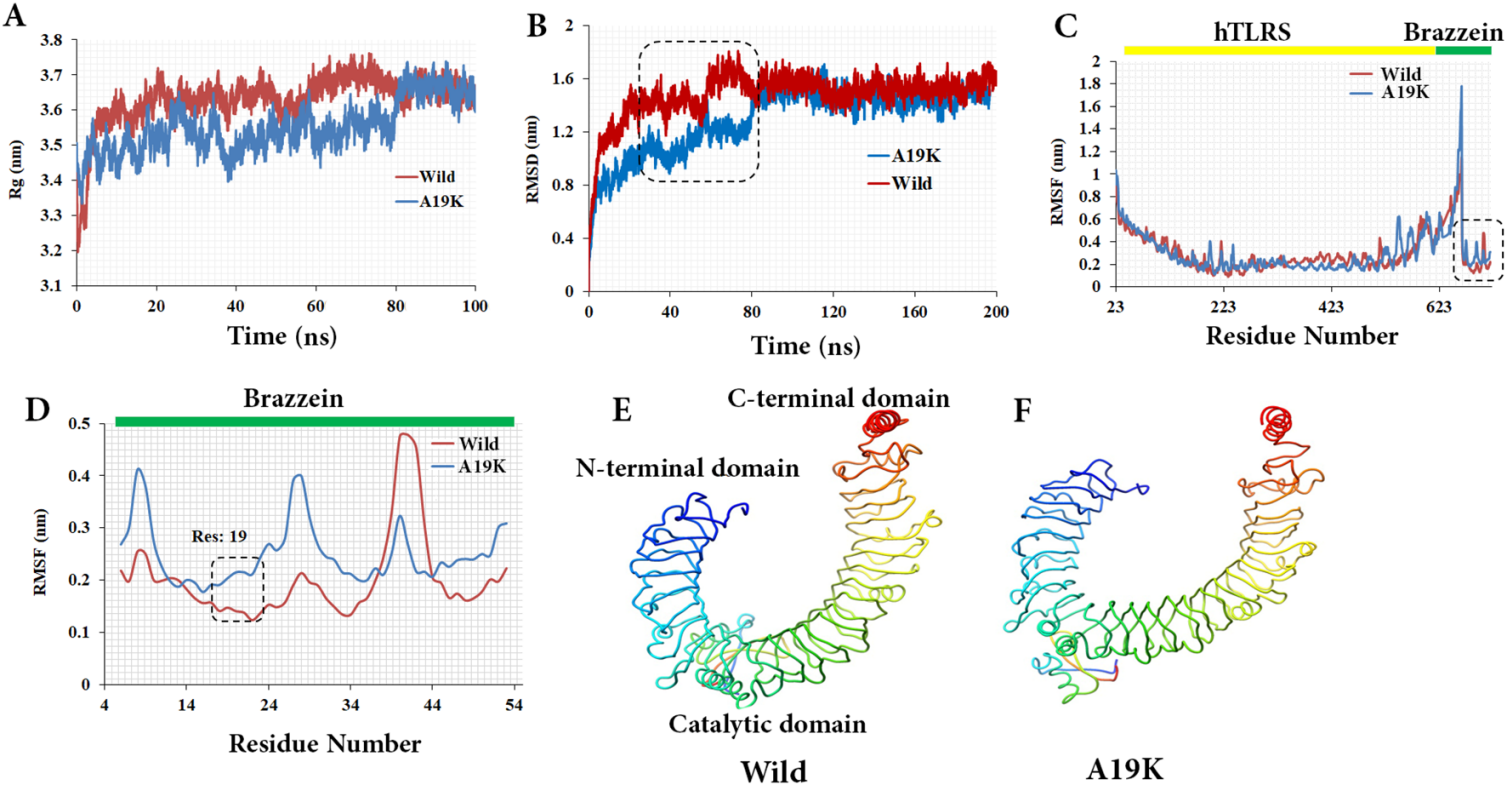
Intrinsic dynamic stability of the modeled hTLR5- WT and hTLR5-p.A19K complex during all-atoms MD simulation of 100 ns. (**A**) Radius of gyration (Rg) plot showing the compactness of trajectory of the two systems over the time scale of 100 ns. (**B**) The backbone RMSD of two MD systems over the time scale of 100 ns (blue: p.A19K-hTLR5 and red: hTLR5-WT complex). Square dashed line is the greatest flexibility in the WT. Bar plot showing the average RMSD for the WT and hotspot mutant forms. (**C**) The residual flexibility is measured by RMSF of Cα-atoms of the hTLR5-Brazzein. Square dashed line shows that the stability of CT end region for WT/hTLR5 was slightly more than p.A19K/hTLR5 complex. (**D**) The RMSF plot of Brazzein during the simulation time containing hotspot mutation. (**E**,**F**) B-factor analysis of the MD simulated WT and p.A19K/hTLR5 complex, respectively.

The Rg values for the protein were plotted against the 100 ns of MD simulation to analyze the compactness, shape, and folding of the overall structure of WT and p.A19K mutant in complex with hTRL5 (Fig. 2A). In fact, the Rg exhibits the compactness and overall dimension of protein or macromolecular complexes. It enlightens the packing of secondary structures into the 3D structure of a protein^24^. The results demonstrated that the Rg of each compound remained stable after 23 ns of MD simulation. The simultaneous changes in the Rg curves of the complex with TLR5 revealed a similar pattern of Rg values although the replacement of residue Ala 19 with Lys caused a higher deviation with an Rg score of 0.15 nm. Regarding the WT, the aggregate Rg increased by more than 0.5 nm. Based on the results of Fig. 2A, the Rg plot fluctuated during 30–80 ns simulation time. It seems that the structure of the complexes may be altered during simulation. Furthermore, the WT-hTLR5 complex had a mean Rg of around 3.63 nm, while a slightly lower Rg of about 3.55 nm was obtained in another, which represents the greater compactness of the system during the 100-ns simulation. The similar Rg for both of the structures suggested the tight packing of the WT-hTLR5 complexes, making the structure relatively stable (Fig. 2A). The Rg curve compression of Brazzein with TLR5 is similar to the RMSD and RMSF parameters, indicating that Brazzein tries to reach internal configuration in hTLR5.

Two MD simulations were performed for WT and p.A19K mutant at 37 °C to compare proteins in terms of structure and dynamic behavior. Additionally, the RMSD values for the backbone atoms of the proteins were monitored relative to the starting structures over the simulation time. As depicted in Fig. 2B, the WT structure reflects a rapid rise to 1.14 nm following the beginning of the simulation run. The substitution of A 19 with K leads to lower fluctuations than the WT (≈ 50 Å) although the greatest flexibility belongs to the WT during the 50–70 ns of running, which is in line with the results of Rg plot after 80 ns. Further, the fluctuations are very small and both trajectories have almost the same values. Therefore, the structures are in their equilibrium states, which are highly correlated with certain interaction energies such as van der Waals force and non-polar desolvation^25^.

The RMSF analysis can be used as a tool to describe local flexibility differences among residues throughout the MD trajectory^26^. It was expected that the catalytic domain of hTLR5, including the residues 23–664 in complex with Brazzein, exerts various structural effects on the different regions of the protein (Fig. 2C,D). The residues had well-known interactions with hTLR5. The peak fluctuation values were 0.5 and 1.8 nm for the WT and p.A19K mutant, respectively. In both complexes, the N-terminal (NT) end was quite flexible than the C-terminal (CT) one (Fig. 2C), while WT-hTLR5 complex possessed a slightly more stable CT end region in comparison with another. The CT end region is a fragment of K15–N20 in the Brazzein structure, which was important for binding to hTLR5 and underwent hotspot mutation. The result is in good agreement with that of Ghanavatian et al.^5,27^. which revealed a region of protein containing position 19 as one of the critical points for interacting with the receptor. Seemingly, any change in this position may alter the stability of the corresponding complex. The B-factors or thermal fluctuations of the complexes modeled from MD simulation were computed to corroborate the results of RMSF analysis. The results of RMSF analysis are perfectly correlated with the B-factor one where the central loops illustrate a high degree of deformity along the NT and CT regions. Furthermore, the PCA was carried out on the MD trajectory during 100 ns to understand the motion changes in both complexes in more details (Fig. 2E,F).

**Figure 3:**
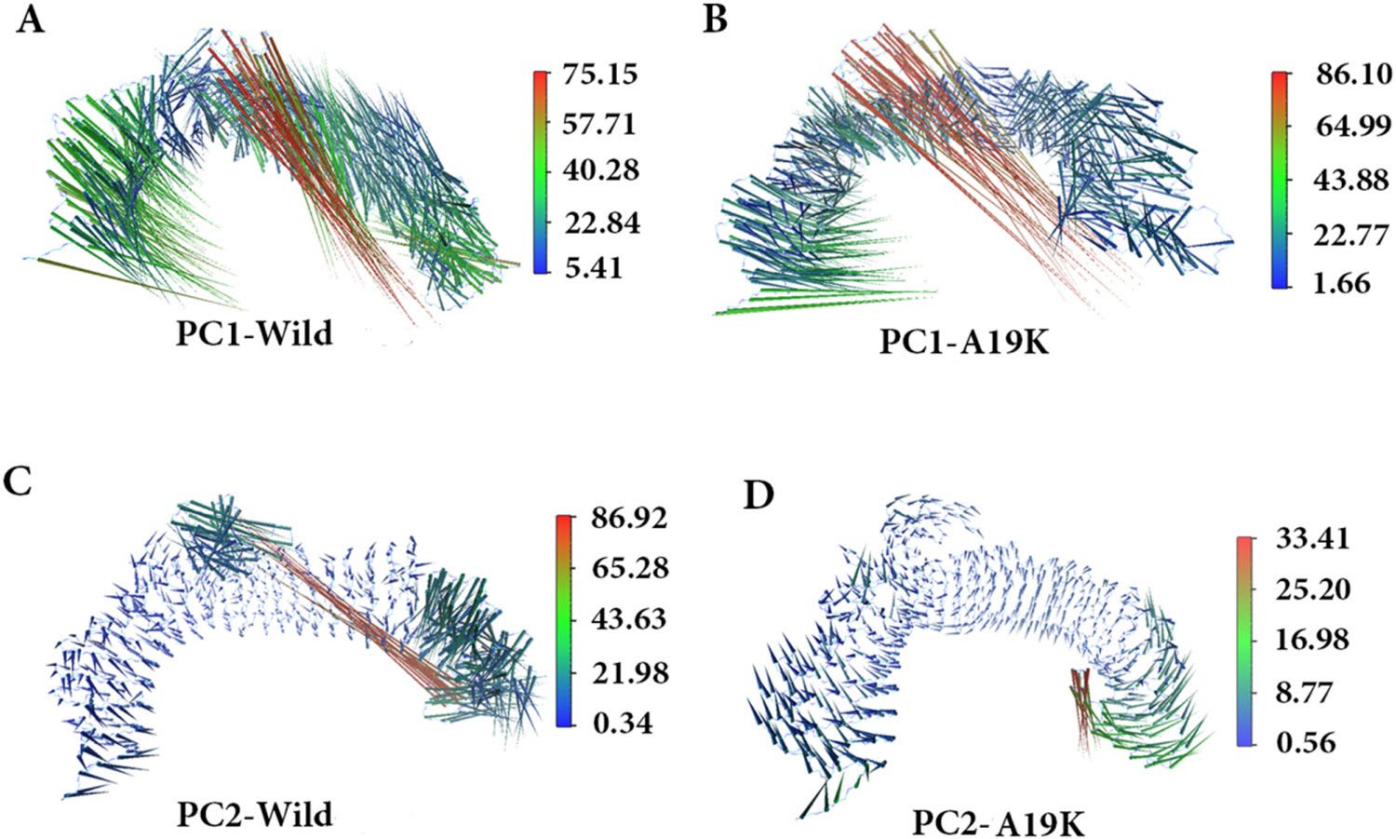
Principal component analysis of Brazzein /hTLR5 complex during the 100 ns of MD. (**A**,**B**) plot displaying the movement of WT and p.A19K/hTLR5 by analyzing the PC1 from PCA. (**C**,**D**) Porcupine plot displaying the movement of WT and p.A19K/hTLR5 by analyzing the PC2 from PCA.

To comprehend the motion directions captured by the two top-ranked principal components (PCs) quantitatively, porcupine plots were generated by using the extreme projections on first (PC1) and second PC (PC2) (Fig. 3). In the cases, the length and direction of arrow denote the strength and direction of motion, respectively. The porcupine plots suggest the high-degree inward motion in the CT and NT regions of WT and p.A19K mutant in PC1. In the case of PC2, inward motion is only observed in WT, while the CT region of p.A19K mutant has outward motion. The results of PCA suggested the terminal end of motion modes, as well as central loops as the major flexible regions in p.A19K-hTLR5 complex, which are completely consistent with those of RMSF and B-factor analysis.

### Essential dynamics

**Figure 4:**
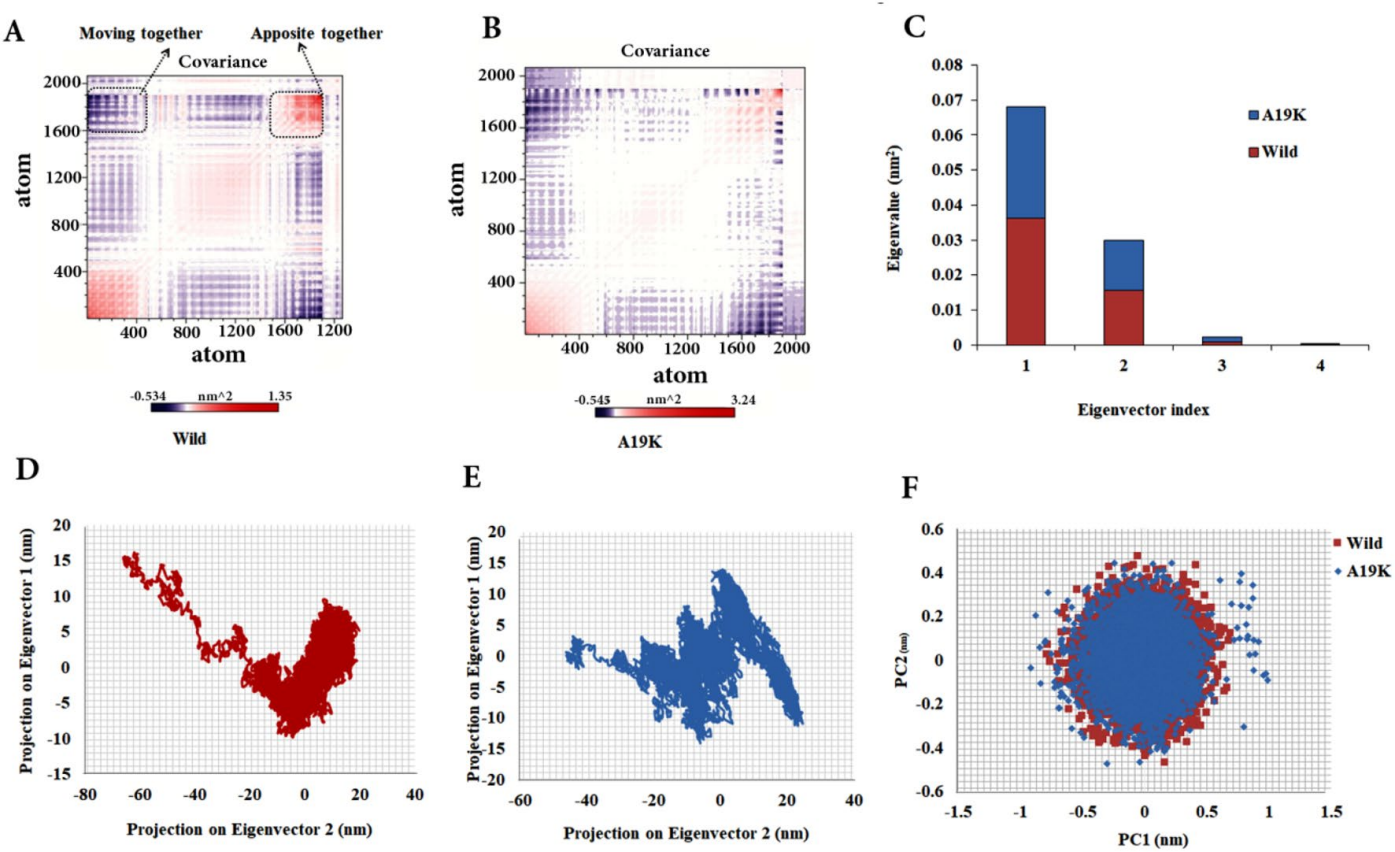
Dynamic effects of the WT and p.A19K variant in the Brazzein /hTLR5 complex. (**A**,**B**) Covariances between WT and mutant residues in complex with hTLR5. (**C**) Bar plot of the eigenvector index versus eigenvalues for the first four eigenvectors of two protein–protein complexes. (**D**,**E**) Cross correlation matrix C-alpha atomic graph and plot in during 100 ns simulation for the wild and the mutant systems. (**F**) PCA scatter plots for the first and second principal components (PC1 and PC2) for WT and p.A19K variant, showing differences between the eigenvectors of the two types.

In the present study, the PCA was employed to describe fluctuation, flexibility, and atomic positional fluctuations for quantifying the main correlated motions of the WT and mutant, as well as identifying major dynamic protein regions (Fig. 4). The covariance matrix and 2D projection of simulations were mathematically assessed for the brazzein-hTLR5 complex by using two PCA eigenvectors during the 100-ns simulation (Fig. 4). In this plot, a positive correlation between two residues reflects a determined motion along the same direction (blue), while a negative one refers to the opposing directions of motion (red)^26^. Based on the results in porcupine curves, the most correlated motions were found in the WT, the majority of which was centered on the interaction between Brazzein and TLR5 (Fig. 4A,B). As shown in Fig. 4, the PC1 and PC2 between interacting residues indicate a twisting motion. The range of motion varies − 0.534 to 1.35 in the WT, while it is larger in the mutant (− 0.545 to 3.24). In general, the blue and red spots are more focused on the WT although they are broadly scattered and less intense in the mutant. In addition, more uncorrelated motions are detected in the mutant one. The results of correlation matrix analysis of the p.A19K mutant represent more scissoring motions along the PC1 axis compared to the WT (Fig. 4B). At the qualitative level, the PCA scatter plots obtained by using the first two PCs (PC1 and PC2) for the both models demonstrate the differences between the two types of eigenvectors (Fig. 4C–E). The calculated eigenvectors are displayed as bars in Fig. 4C, which clearly exhibits that the first four eigenvectors account for more than 80% of the collective motions of Cα protein atoms. Further, the first two PCs possess the highest range of motion. The values related to PC1 and PC2 are respectively equal to 0.03174 and 0.01554 nm^2^ in the WT, as well as 0.03628 and 0.01441 nm^2^ in the p.A19K mutant. Furthermore, the comparison of the values reflects a greater range of motion in the simulation of p.A19K mutant. The scatter plot of PC1 against PC2 in phase illustrates the occupation of less conformational space by WT-hTLR5 complex than the p.A19K-hTLR5 one. Finally, p.A19K-hTLR5 complex occupies wider conformational space than the experimental complex due to the high degree of flexibility (Fig. 4F).

### Interaction of Brazzein with hTLR5

**Figure 5:**
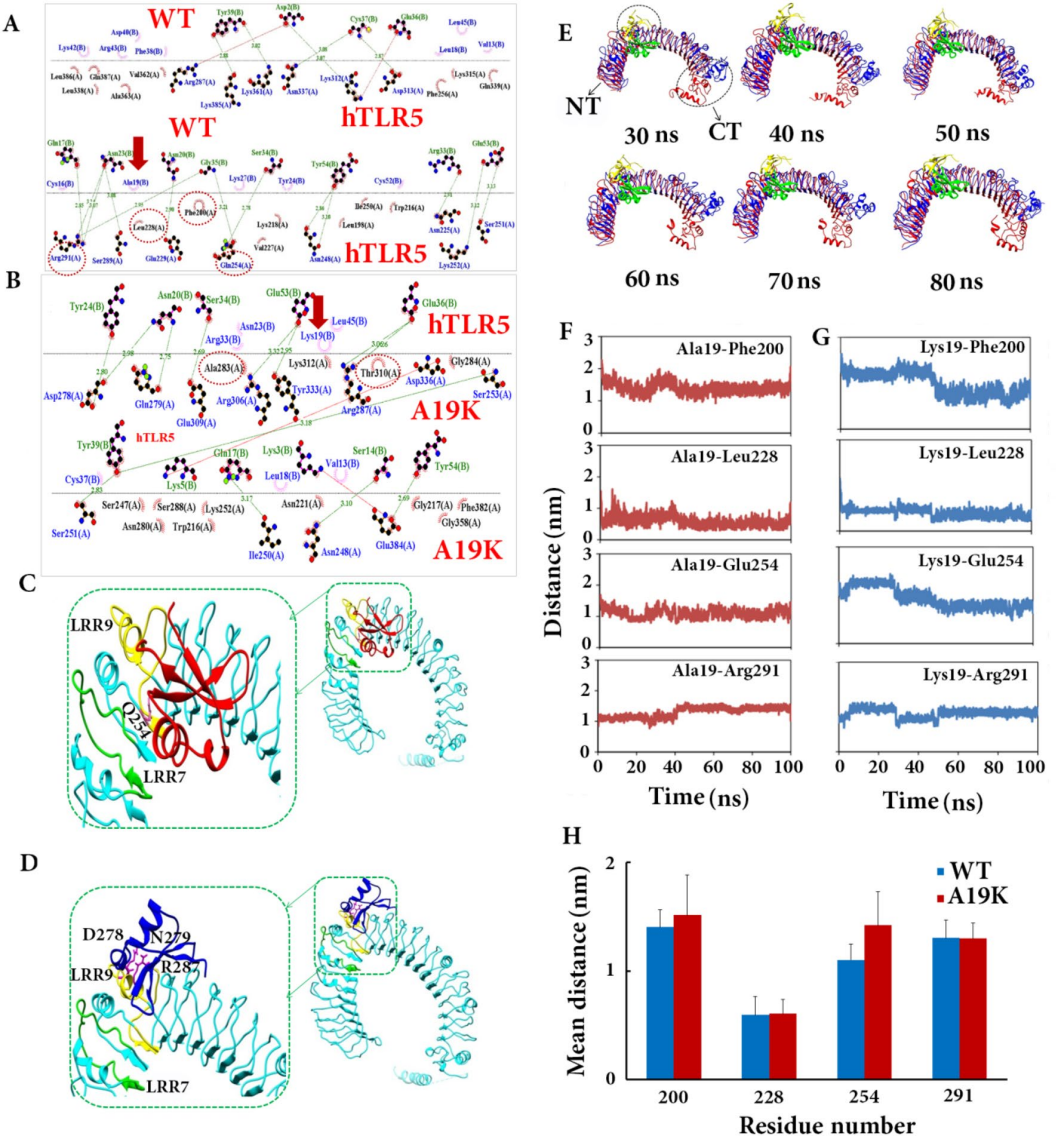
Intermolecular contact analysis of Brazzein and hTLR5 displaying various non-bonded interactions. (**A**) Contact analysis of hTLR5-WT complex obtained after MD. (**B**) Contact analysis of hTLR5-p.A19K complex obtained after MD. The hydrogen bonds are shown in dashed lines while the interacting residues are shown in ball and stick representation. The other hydrophobic contacts are shown in semi-circles. Dotted circle shows all residue which interact with Ala 19 in WT-type and p.A19K as hotspot mutation in Brazzein. (**C**) The superimpose of WT of Brazzein (red color) and hTLR5 (cyan color). LRR7 and LRR9 domains of hTLR5 have been shown in green and yellow colors, respectively which Q254 of Brazzein is located in the LRR9 domain of hTL5. (**D**) The superimpose of p.A19K mutant of Brazzein (blue color) and hTLR5 (cyan color). LRR7 and LRR9 domains of hTLR5 have been shown in green and yellow colors, respectively which D278, N279, and R287 of Brazzein are located in the LRR9 domain of hTLR5. (**E**) The WT structure (green color) superimposed on either the p.A19K mutant (yellow color) of Brazzein in complex with hTLR5 (WT in blue and mutant in red colors) to compare the level of fluctuation in the LRR and CT domains during the simulation time. The p.A19K mutant showed a clear structural change from 30 to 80 ns. (**F**,**G**) Inter-atomic distance profile of the important hydrogen bond forming amino acid pairs in hTLR5-Brazzein complex using the MD trajectory. (**H**) The average distance between the interacting pairs of atoms from Brazzein and hTLR5 are summarized in bar plot. The average distance of two residues i.e. Phe 200 and Glu 254 was increased in the presence of hotspot mutation.

To gain deep insights into the interactions mediated by Brazzein, the final conformations of both the complex systems were utilized for interaction analysis in LigPlot+. In the binding site, 18 hydrogen bonds (mean distance of ~ 3 Å) were formed between the residues Asp 2, Gln 17, Asn 20, Asn 23, Arg 33, Ser 34, Gly 35, Cys 37, Tyr 39, Glu 53, and Tyr 54 in the WT with the residues Asn 225, Glu 229, Asn 248, Ser 251, Lys 252, Gln 254, Ser 289, Arg 291, Lys 312, Asp 313, Asn 337, Lys 361, and Lys 385 in hTLR5 (Gln 254 is in the LRR9 domain) (Fig. 5A). Additionally, the amino acids of Ser 14, Gln 17, Asn 20, Tyr 24, Ser 34, Glu 36, Tyr 39, Glu 53, and Tyr 54 in p.A19K mutant acted as interacting residues in 13 hydrogen bonding (mean distance of ~ 2.98 Å) with residues Asn 248, Ile 250, Ser 251, Ser 253, Asp 278, Gln 279, Arg 287, Arg 306, Glu 309, Tyr 333, and Glu 384 in hTLR5 (Asp 278, Gln 279, and Arg 287 are considered as the LRR9 of hTLR5) (Fig. 5B). According to Bell et al.^28^, hTLR5 binding site is located in the LRR7 region (197–226) and LRR9 (254–288) can affect the response to Brazzein. This domain coupled the large domain modeled in this study to the catalytic one of Brazzein. In the WT-hTLR5 complex, the four residues Phe 200, Leu 228, Glu 254, and Arg 291 in the catalytic domain of hTLR5 hydrophobically interacted with the Ala 19 of Brazzein. However, two residues like Ala 283 and Thr 310 were involved in the hydrophobic interaction with hTLR5 following the replacement of residue Ala 19 with Lys. Further, major differences were observed with respect to the number of hydrophobic contacts so that the WT-hTLR5 complex had more electrostatic and pi-alkyl contacts compared with another (Fig. 5A–D).

Given the high thermal stability and low chemical stability of Brazzein reported in the previous studies, the presence of disulfide bonds profoundly influenced the structural integrity of the protein^5^. In summary, the antithetical properties of p.A19K mutants on the protein activity revealed that the presence of charged residue at this position may exert a profound effect on the interaction between protein and corresponding receptor. Furthermore, the number of the positively-charged amino acids in the WT can enhance the interaction strength toward increasing the sweetness of protein. Seemingly, residues Ser, Gln, Asn, Tyr, and Glu in the p.A19K mutants were directly implicated in forming hydrogen bonds with other residues and polar water molecules. However, the interactions distorted β-strands, and decreased the stability of β-sheets or overall conformational stability of protein. The above-mentioned issues and gene expression results introduced local flexibility and positive charge at the protein surface as the more important factors affecting the protein interaction with the G-protein-coupled human sweet receptor. Therefore, this mutation destabilized the CT of hTLR5 binding to Brazzein. The results are in line with those of the previous studies^5^.

Figure 5E demonstrates the WT structure (green) superimposed on either the p.A19K mutant (yellow) in complex with hTLR5 (WT in blue and mutant in red colors) to compare fluctuation level in the LRR and CT domains during the simulation. As depicted, the p.A19K mutant structurally changes over 30–80 ns, and exhibits more flexible structure compared to the WT, which are confirmed by using the results of RMSD curve.

The results of detailed structural analysis indicated the presence of more H-bonds in the WT than the p.A19K, which may alter the conformation of several overlying residues at the LRR and CT domains.

Regarding both the complexes, the interatomic distance between the crucial interacting pairs was measured from the MD trajectories (Fig. 5F,G). The mean distance between the interacting pairs of atoms in Brazzein and hTLR5 are outlined in Fig. 5H. The distance profile represented the continuous participation of the important amino acid residues in the molecular recognition process of Brazzein by hTLR5. The mean distance of the residues Phe 200 and Glu 254 elevated in the presence of hotspot mutation. Generally, the MD simulation of both hTLR5 complexes revealed a stronger interaction between the WT receptor and hTLR5.

### Induction of instability in free energy landscape (FEL) by the p.A19K mutant

**Figure 6:**
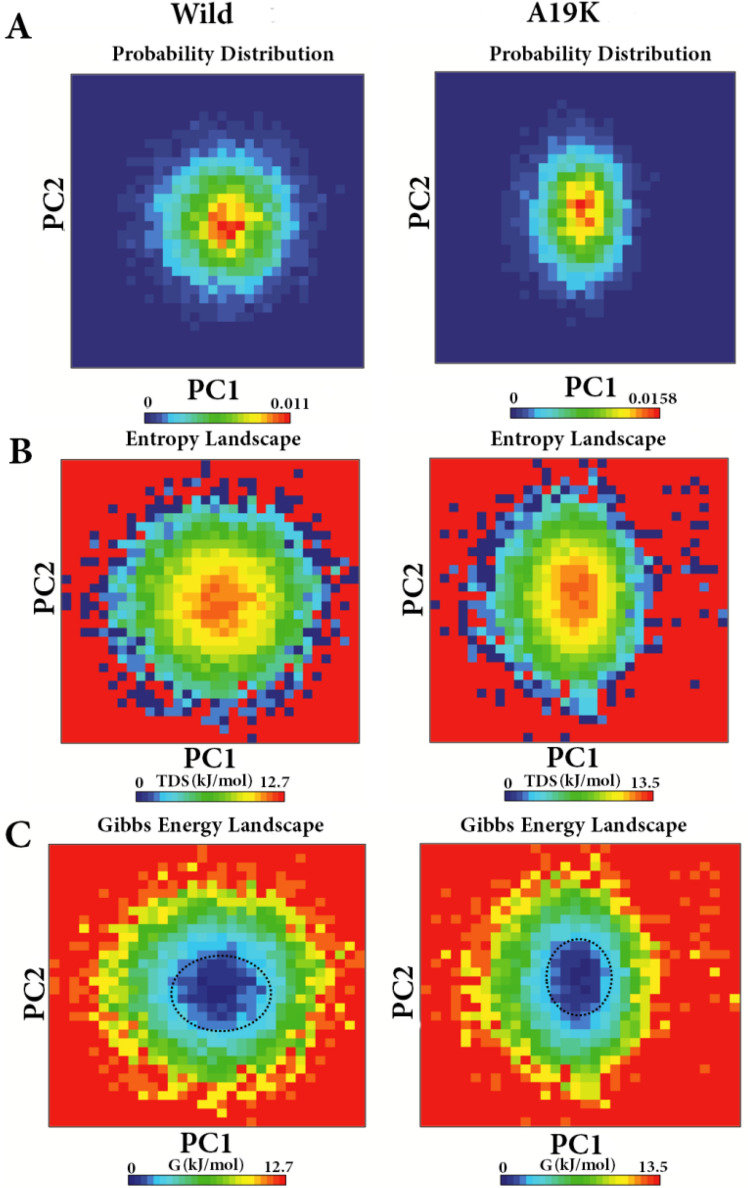
Probability distribution modification of the first two eigenvectors and fluctuation and free energy landscape of the WT and p.A19K forms of the Brazzein /hTLR5 complex. (**A**) Probability distribution modification of the first two eigenvectors and fluctuation of the WT and p.A19K forms. The dark blue and red colors indicate the lowest and the highest probability motions, respectively. (**B**) The entropy of the structure of the WT complex in comparison with the mutant forms. (**C**) Free energy landscape for the WT and p.A19K forms of the complex.

According to Fig. 6A, most motions in the first PC were attributed to the p.A19K mutant, which is consistent with the results of the PCA scatter which reflected the probability distribution of the first two eigenvectors and fluctuations of Brazzein mutant. The comparison between the conformational entropy of the complexes based on the PC1 and PC2 produced from the PCA indicated the highest entropy in the p.A19K mutant (Fig. 6B). As demonstrated in Fig. 6C, the minimum energy clusters are highlighted with black dotted circles. The results of the analysis exhibited a centralized energy distribution, suggesting the overall conformational stability of this system. Thus, the mutation of the residue, p.A19K, influenced the overall conformational stability of the brazzein-hTLR5 complex. The variations in the cumulative motion correlation profile for the p.A19K-hTLR5 complex revealed the relative differences in both landscapes.

### Effect of Brazzein on the MCF-7 cell viability

**Figure 7:**
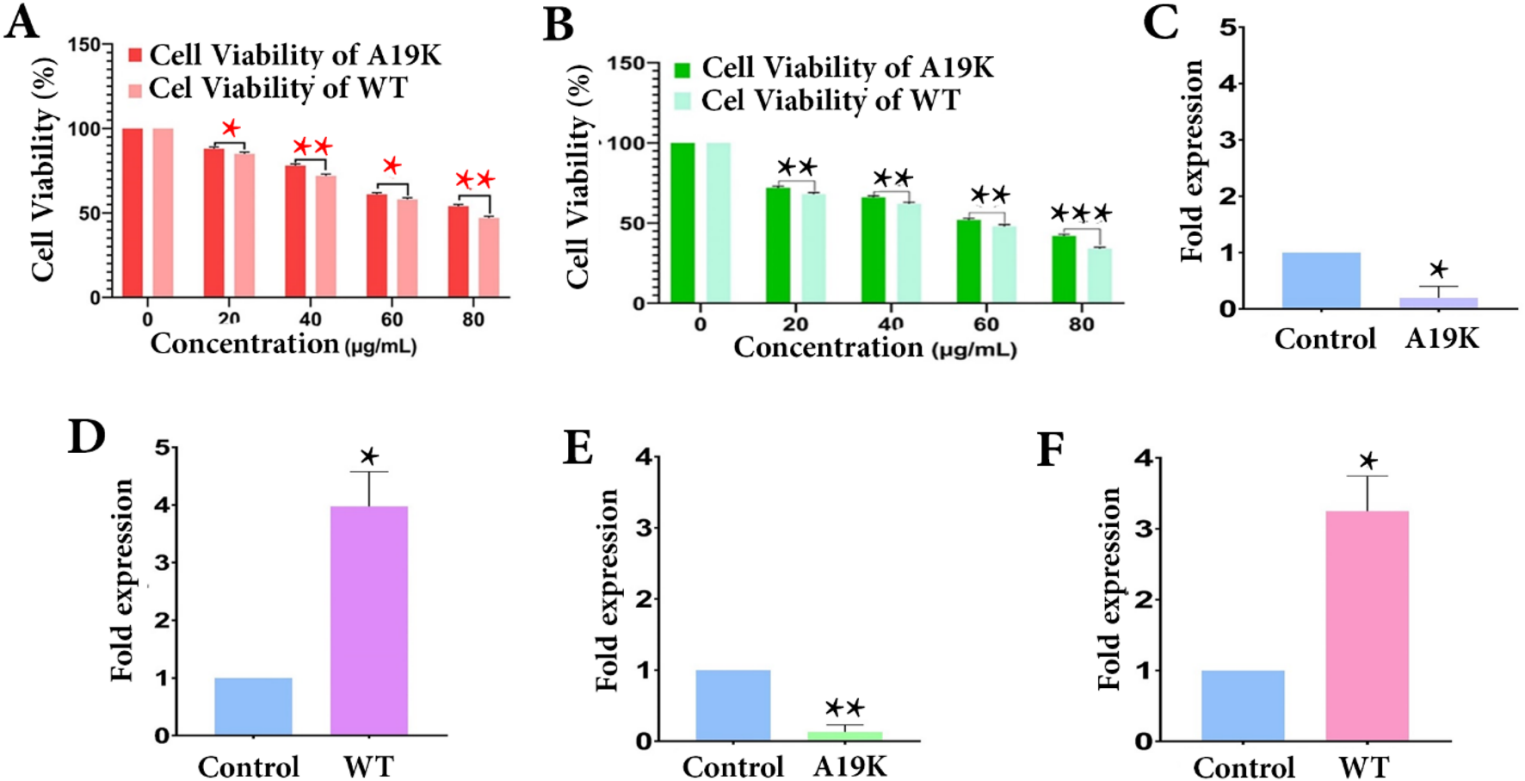
Effect of WT and p.A19K Brazzein on MCF-7 cell line viability. (**A**,**B**) The cells are treated with various concentrations (20, 40, 60, 80 μg/ml) of Brazzein and p.A19K mutant for 48 h and 72 h, respectively. Data values are expressed as % of control values. Significant change (*P < 0.05, **0.05 < P < 0.01 and ***P < 0.001) in comparison with control group). Real-time PCR analysis for *TNF-α* and *MAP1S* genes in MCF-7 cells. The values put onto each graph represent the relative fold change calculated by calibrating the ΔΔCt data *TNF-α* (**C**,**D**) and *MAP1S* (**E**,**F**) gene expressions, respectively. Error bars indicate mean ± standard deviation (SD). The significant level was define as *P ≤ 0.05 and **0.05 < P < 0.01.

n this study, the cells were incubated with the various concentrations of the WT and p.A19K mutant for 48 and 72 h. Based on the results, the cell viability increased in WT (85, 72, 58, and 47%) and p.A19K mutant (88, 78, 61, and 54%) after incubating for 48 h (Fig. 7A). Therefore, 80 μg/ml level of Brazzein was better in the WT than the p.A19K mutant. A comparison between the WT protein and p.A19K mutant treated for 72 h is provided in Fig. 7B. The WT at 20, 40, 60, and 80 μg/ml amounts reduced cell viability by 68, 62, 48, and 34%, respectively. However, 72, 66, 52, and 42% decline was observed following the incubation with the same concentrations of p.A19K mutant, respectively. Given that both the WT and p.A19K mutant had IC50 of 60 μg/ml level in the 72-h treatment, this concentration was used for further evaluation.

### Expression of ***TNF-α*** and ***MAP1S*** genes

The TLR5 signals through the MYD88 pathway, which drives the production of inflammatory cytokines such as tumor necrosis factor-alpha (*TNF-α*) and interleukin-12 (IL-12)^29^. The antitumor mechanisms of hTLR5 signaling in breast cancer cells suggest *MAP1S* as an important autophagic adapter, which is associated with tumor suppression via autophagy regulation. *MAP1S* level promotes in response to flagellin stimulation in the MCF-7 cells^16^.

Compared to the control group, *TNF-α* gene was significantly less and more expressed in the p.A19K mutant (P < 0.05) and WT, respectively (Fig. 7C,D). In terms of *MAP1S* gene expression, the results reflected a significantly decrease in the p.A19K mutant (P < 0.01) and an enhancement in the WT (P < 0.05) compared to the control (Fig. 7E,F).

## Discussion

This study provided a molecular rationale for understanding the conformational changes resulted from binding Brazzein to hTLR5 in the breast cancer cell line better. It presented the binding mode of Brazzein, a sweet protein, to PRRs for the first time. Despite the possible direct effect of the mutant on binding properties, the study considered that it is more likely to affect the conformational change of hTLR5 required to activate second messenger signaling within the cell. Similarly, the point mutation in Brazzein failed to act as presumed. In other words, the mutation did not cause autophagy by reducing gene expression. However, the WT protein led to the autophagy of breast cancer cells by elevating gene expression and activating the TLR5 pathway, which may be a good candidate as an anticancer peptide. Both the p.A19K mutant and WT similarly influenced breast cancer proliferation, while the p.A19K mutant did not enhance the hTLR5 signaling genes. Further, the strong correlation between the computational estimations and gene expression obtained by RT-PCR through cell-based responses upon exposure to Brazzein and hTLR5 strengthens the idea of using molecular docking and dynamics for designing and predicting the stability of their interaction. Furthermore, the WT can be introduced as the hTLR5 agonist, as well as can better respond to hTLR5 than the p.A19K, which is in line with the modeling results. It is proposed that future studies focus on the experimental assessment of this result through using the final Brazzein-receptor model to design the new mutants of the protein.

## Materials and methods

### Materials

Recombinant *E. coli* strain Shuffle^®^T7 was utilized for gene expression in the wild type (WT) and p.A19K mutant of Brazzein^5^. In addition, MCF-7 human breast cancer cell lines were obtained from the Pasteur Institute of Iran. DMEM-high-glucose, fetal bovine serum (FBS), trypsin-EDTA, antibiotic (Pen-Strep), and phosphate buffered saline (PBS) were purchased from Inoclon (Iran). Further, MTT was provided by Merck (Germany), and RNAse extraction and cDNA synthesis kits were acquired from GeneAll (Korean) and Pishgam (IRAN), respectively. The other chemicals applied in this study were commercially available with the highest analytical grade.

### Homological modeling

The molecular docking analysis was employed to examine the interaction between Brazzein and hTLR5 molecules. The 3D structure of the WT (PDB code: 2LY5) as a ligand^30^ and human TLR5 (PDB code: 3J0A) as a receptor^31^ was extracted from the protein data bank (http://rcsb.org/). Furthermore, a mutation of alanine (Ala) to lysine (Lys) at the 19th position was generated in silico in the WT crystal structure using Modeller 9.20 software^32^. The general stereochemical quality of the final modeled protein structure was evaluated based on the parameters of Verify 3D, Z-DOPE, and RMSD obtained using SAVES (https://saves.mbi.ucla.edu/) and ModEval servers, (https://modbase.compbio.ucsf.edu/evaluation/) and SpdbViewer software, respectively^33,34^. The structures were also analyzed by the Ramachandran Plot Server (https://zlab.umassmed.edu/bu/rama/) to be sure that the selected structure has the minimum questionable positions, as compared with the other structures^35^.

### Protein–protein interaction (PPI)

The protein–protein interactions (PPI) are known as the midpoint for all of the biochemical pathways involved in biological functions. The interactions were assessed in high ambiguity‐driven protein–protein docking (HADDOCK) 2.2 program^34^. The protein structural calculations were guided by defining active residues based on the previous report^28^, followed by selecting the structures with the least HADDOCK score and best binding site. Then, the program for automatically plotting protein–protein interactions (Ligplot-Dimplot, v 2.2.5) was applied to determine intermolecular contacts such as hydrogen bonds and non-bonded ones^36^. Finally, the 3D models of docking and molecular dynamics (MD) results were analyzed by using Chimera 1.13.1 software^36^.

### MD simulation

The computational studies on the biological macromolecules, provides a plethora of various data types as the output that can be more analyzed and processed^37,38^.

In this study, MD simulation was performed to evaluate the motions and fluctuations of two systems under the effect of the internal and external forces generated by hotspot mutant residue in Brazzein. Additionally, 100-ns simulations of the p.A19K mutant, detecting the conformational changes in the functional binding domain of Brazzein in complex with hTLR5, were independently created by using the GROMACS MD package (v 2021.2)^39^ in the CentOS Linux system, and implemented with the GROMOS96 force field (v 43a1). This MD protocol has been previously described in the literature^40^. Briefly, simulated proteins were solvated in a 1.0 nm cubic box in the presence of Na^+^ and Cl^−^ ions through using SPC/E water model. The number of solvent molecules and total charge added to each system were 66,030 and 10 ions for wild type and 75,034 solvent and 10 ions for mutant. All of the covalent bonds to hydrogen atoms were constrained based on the SHAKE algorithm. Further, electrostatic interactions were calculated according to the particle-mesh Ewald (PME) algorithm^41^, and pre-equilibration was carried out with approximately 50,000 steps of steepest-descent minimization. After minimizing energy, the system was equilibrated by using the 100-ps simulation of a NVT (canonical) followed by a NPT simulation (P = 1 bar) and gradual heating from 310 K. The Berendsen thermostat^42^ algorithm was utilized to maintain constant temperature and pressure^43^. Furthermore, 50,000 steps of minimization and 100 ns of unrestrained MD were performed. Then, short-range electrostatic and van der Waals interactions were computed with a distance cut-off of 1.0 nm^18,44,45^. After equilibration, coordinates were saved every 2 ps during the sampling process. The conformations generated from the simulations were used in further analyses. Finally, the coordinates and periodic corrections were examined by installing trjconv tools in the GROMACS software package. We have not capped the N- and C-terminals during simulation, as terminal residues are seen in the structure and are present in the PDB coordinates.

### Trajectory analysis

The comparative equilibration was determined from a flattening of the radius of gyration (Rg), as well as root mean square deviation (RMSD), residue-based root mean square fluctuation (RMSF), secondary structure modifications, between-atom distances, free-energy landscape (FEL), entropy, principal component analysis (PCA)^46^, and eigenvectors (Evs) over time after a 100-ns interval for WT and mutant model.

### Protein preparation (expression and purification)

In this step, the *E. coli* strain Shuffle^®^ T7 containing the genes of the WT and p.A19K mutant was incubated in 5 ml of the luria broth (LB) medium supplied with kanamycin (50 mg/ml) overnight at 30 °C and regularly shaken at 180 rpm. In addition, 200 μl of pre-cultures was diluted in 200 ml of the TB incorporating kanamycin (50 mg/ml) and incubated at 30 °C until reaching the absorbance of almost 0.8–1.0 at 600 nm, to which 0.5 mM IPTG was added for 2 h. The proteins were purified by using a Ni–NTA protein purification system, the His-tag of which was removed with CNBr. Further, an Amicon-ultra 3 kDa column was applied for concentration and buffer replacement with PBS. The quality of purification was evaluated by using 16% tris-tricine SDS-PAGE^5,41^, and a 0.22-μm CA syringe filter (MS, USA) was used to sterilize the protein-containing solutions.

### Cell culture and treatment

The MCF-7 cell lines were cultured in the complete Dulbecco’s Modified Eagle’s Medium (DMEM) supplemented with 10% FBS (Gibco-BRL, Life technology, Paisley, Scotland) and 2% penicillin–streptomycin (pen-strep). Then, they were incubated at 37 °C under a humidified atmosphere (5% CO_2_-95% air) and utilized in all experiments.

### MTT assay for cell viability assessment

The MCF-7 cell lines were seeded into a 96-well plate (10,000 cells/well) in 100 μl of complete DMEM, exposed to the WT and p.A19K mutant at various concentrations (20, 40, 60, and 80 μg/ml), and incubated for 48 and 72 h. After the treatment period, 0.5 mg/ml MTT salt (Sigma-Aldrich, USA) was poured per well, followed by incubating the cells at 37 °C in 5% CO_2_-95% air for 3.30–4 h. The medium was displaced by adding 100 μl of detergent reagent (DMSO) into each well for 10 min to solubilize the colored crystals. Furthermore, microplate readers (BioTek, Elx800, USA) were applied to read absorbance at 490 nm spectrophotometrically. The cell viability rate was obtained as the percentage of reduced MTT by considering the absorbance of control cells as 100%^47^.

### RNA extraction

Total RNA was isolated from the treated cells by using Rneasy kit (RNAse extraction kit, GeneAll, Korean) based on the manufacturer’s recommendation. Additionally, the concentration of RNA samples was determined by measuring optical density at 260 nm and their quality was confirmed by detecting 18S and 28S bands on agarose gel electrophoresis. The RNA samples were incubated with Dnase at room temperature for 15 min to remove residual DNA contamination.

### cDNA synthesis

The total RNA from each sample was used to form cDNA with oligo (dT) primers according to the manufacturer’s protocol (cDNA synthesis kit, Pishgam, IRAN).

### Oligonucleotide primers

This study utilized *TNF-α*, *MAP1S*, and *β-actin* (housekeeping gene). All of the primers were designed with Gene Runner, the sequences of which are presented in Table 4.

**Table 4:** Primers used for REAL-time PCR.

Gene	Primer	Sequence (5′ → 3′)	Length (bp)
MAP1S	Forward	GGAGTGAGCCCAGTGAGAAG	20
	Reverse	CAGGTAGACCGGGGACTTG	19
TNF-α	Forward	CGAGTGACAAGCCTGTAGC	19
	Reverse	GGTGTGGGTGAGGAGCACAT	20
β-actin	Forward	TGAAGATCAAGATCATTG	18
	Reverse	TAACGCAACTAAGTCATA	18

### Molecular analysis: quantitative real-time polymerase chain reaction assay

The *TNF-α* and *MAP1S* genes were evaluated using the housekeeping gene of *β-actin*. Further, the primers were previously checked through employing conventional real-time polymerase chain reaction (RT-PCR) and agarose gel electrophoresis (1.5%). The quantitative RT-PCR (qRT-PCR) was performed on a final volume of 20 μl containing 4 μl of Eva Green qPCR Mix, 1 μl of cDNA, and 1 μl of each of forward and reverse primers (Table 4). The cycling protocol involved an initial denaturation step at 95 °C for 15 s, followed by 40 cycles of denaturation at 95 °C for 15 s, annealing at 52 °C for 15 s, and extension at 72 °C for 15 s. Furthermore, the CT values obtained based on the CT relative quantification method were normalized for endogenous^48^. The fold-change in gene expression was computed using the melt curve approach and normalized to β-actin. Ultimately, the relative gene expression levels were calculated with reference to the control^48^.

### Statistical analysis

The data were analyzed through implementing one-way ANOVA, followed by Tukey's post-hoc test for comparing the mean of each group and an unpaired t-test between groups by using the Prism software (v 9.00) for Windows (GraphPad Software, RRID:SCR_002798, La Jolla, CA) for curve generation and statistical analysis. The data were expressed as mean ± the standard error of the mean (SEM) and p values less than 0.05 were considered as statistically significant difference. Each experiment was repeated in triplicate.

## Data Availability

The datasets of the 3D structure of the WT as a ligand and human TLR5 used during the current study are available in the PDB code: 2LY5 and PDB code: 3J0A, respectively (http://rcsb.org/). The general stereochemical quality of the final modeled protein structure was analyzed based on the parameters of Verify 3D, Z-DOPE, and RMSD obtained using SAVES (https://saves.mbi.ucla.edu/) and ModEval servers, (https://modbase.compbio.ucsf.edu/evaluation/) and SpdbViewer software, respectively.
